# The Substantial First Impact of Bottom Fishing on Rare Biodiversity Hotspots: A Dilemma for Evidence-Based Conservation

**DOI:** 10.1371/journal.pone.0069904

**Published:** 2013-08-14

**Authors:** Robert Cook, Jose M. Fariñas-Franco, Fiona R. Gell, Rohan H. F. Holt, Terry Holt, Charles Lindenbaum, Joanne S. Porter, Ray Seed, Lucie R. Skates, Thomas B. Stringell, William G. Sanderson

**Affiliations:** 1 School of Ocean Sciences, Bangor University, Menai Bridge, Anglesey, United Kingdom; 2 Fisheries Directorate, Department of Environment Food and Agriculture, Isle of Man Government, St John's, Isle of Man, United Kingdom; 3 Natural Resources Wales, Bangor, Gwynedd, United Kingdom; 4 Center for Marine and Coastal Studies Ltd. (CMACS), Port Erin, Isle of Man, United Kingdom; 5 School of Life Sciences, Heriot-Watt University, Edinburgh, United Kingdom; National Institute of Water & Atmospheric Research, New Zealand

## Abstract

This study describes the impact of the first passage of two types of bottom-towed fishing gear on rare protected shellfish-reefs formed by the horse mussel *Modiolus modiolus* (L.). One of the study sites was trawled and the other was scallop-dredged. Divers collected HD video imagery of epifauna from quadrats at the two study sites and directed infaunal samples from one site.

The total number of epifaunal organisms was significantly reduced following a single pass of a trawl (90%) or scallop dredge (59%), as was the diversity of the associated community and the total number of *M. modiolus* at the trawled site. At both sites declines in anthozoans, hydrozoans, bivalves, echinoderms and ascidians accounted for most of the change. A year later, no recovery was evident at the trawled site and significantly fewer infaunal taxa (polychaetes, malacostracans, bivalves and ophuroids) were recorded in the trawl track.

The severity of the two types of impact reflected the undisturbed status of the habitats compared to previous studies. As a ‘priority habitat’ the nature of the impacts described on *M. modiolus* communities are important to the development of conservation management policy and indicators of condition in Marine Protected Areas (EU Habitats Directive) as well as indicators of ‘Good Environmental Status’ under the European Union Marine Strategy Framework Directive.

Conservation managers are under pressure to support decisions with good quality evidence. Elsewhere, indirect studies have shown declines of *M. modiolus* biogenic communities in fishing grounds. However, given the protected status of the rare habitat, premeditated demonstration of direct impact is unethical or illegal in Marine Protected Areas. This study therefore provides a unique opportunity to investigate the impact from fishing gear whilst at the same time reflecting on the dilemma of evidence-based conservation management.

## Introduction

Shellfish reefs are “one of, if not the most imperilled marine habitats on earth” [1]. The loss of 85% of the world's oyster reefs can be estimated from fisheries and other records [2], but declines in non-target shellfish reefs are harder to quantify. Horse mussels (*Modiolus modiolus* Linnaeus) are a non-target species in much of their range and widespread in the northern Atlantic and Pacific Oceans. Dense beds of *M. modiolus* are biogenic reefs [3] and have a more limited known distribution in the White Sea, Bay of Fundy, the Irish Sea, Scotland, Scandinavia and Iceland [4]–[8]. In common with many other types of biogenic shellfish reefs, those formed by *M. modiolus* are known to be threatened and declining [9].

In open-coast locations with moderate to high tidal flow, *M. modiolus* reefs can form long-lived structures up to 3m above the surrounding seabed [4], [10]–[12]. These habitats create high levels of physical complexity where clumps of dense *M. modiolus* provide substrata for an epifaunal community whilst the spaces between mussels accumulate sediment which supports a rich crevice and infauna of 200–300 species, at densities exceeding 22,000 individuals m^−2^ [13], [14]. Tide-swept horse mussel reefs have therefore been identified as rare biodiversity hotspots compared to surrounding habitats, and networks of Marine Protected Areas (MPAs) are under development to support these and other habitats through international and national legislation (EC Habitats and Species Directive; Marine (Scotland) Act 2010; see also [12]). The maintenance of these so called ‘Priority Habitats’ [15] will also contribute to the achievement of ‘Good Environmental Status’ (GES) under the European Union (EU) Marine Strategy Framework Directive (MSFD; 2008/56/EC) such that indicators of their status are under consideration for development [15].

In the 21^st^ century significant concerns have been raised that conservation practice is sometimes based upon anecdote and myth rather than systematic appraisal of evidence [16]. Systematic review has therefore been proposed to support “evidence-based conservation” [16], [17], and sources of evidence used by conservation managers have been increasingly scrutinised [18]. At the same time the impacts of fisheries on the seabed, and by implication the necessity to manage them, have been challenged by stakeholders on the basis of a lack of scientific evidence (see rebuttals by [19]–[21]). Nevertheless, proponents of evidence-based conservation accept that management decisions still need to be made in the absence of good quality evidence [17] and in a marine context some have called for a “reversal of the burden of proof” in management decisions [22].

*M. modiolus* reefs and their associated communities have been found to decline in areas subjected to bottom-towed fishing gear [23]–[25], as have oyster reefs [26]. Furthermore, where *M. modiolus* has been targeted as bait for cod, it has declined and not recovered [27]. The direct effects of bottom-towed fishing gear on sparse *M. modiolus* individuals has been shown [28] but where it occurs in high densities and forms reefs the direct impact of an individual pass of fishing gear has not been described. Indeed, the majority of direct impact studies of trawling and scallop dredging are from soft sediment and gravel communities [21], [29]–[31] while those on complex, temperate biogenic habitats are rare [32].

Habitat rarity can prohibit elegant experimental approaches to support sensitive management [33], but providing the impact evidence - base may also be unethical or illegal if it is necessary to willingly damage a habitat or species in a protected area. In the present study, benthic marks attributed to the single passage of two types of bottom fishing gear were identified during routine monitoring operations on *M. modiolus* reefs. This provided a unique opportunity to investigate, directly, the scale of the epifaunal and infaunal impact under a null model. The study also provided an opportunity to reflect on the consequences of the absence of this kind of information to the conservation manager.

## Materials and Methods

No permits were required for the described study, which complied with all relevant regulations. Protected habitats were sampled in full consultation and collaboration with statutory conservation authorities (Countryside Council for Wales and Isle of Man Government).

### Site information

**Figure 1:**
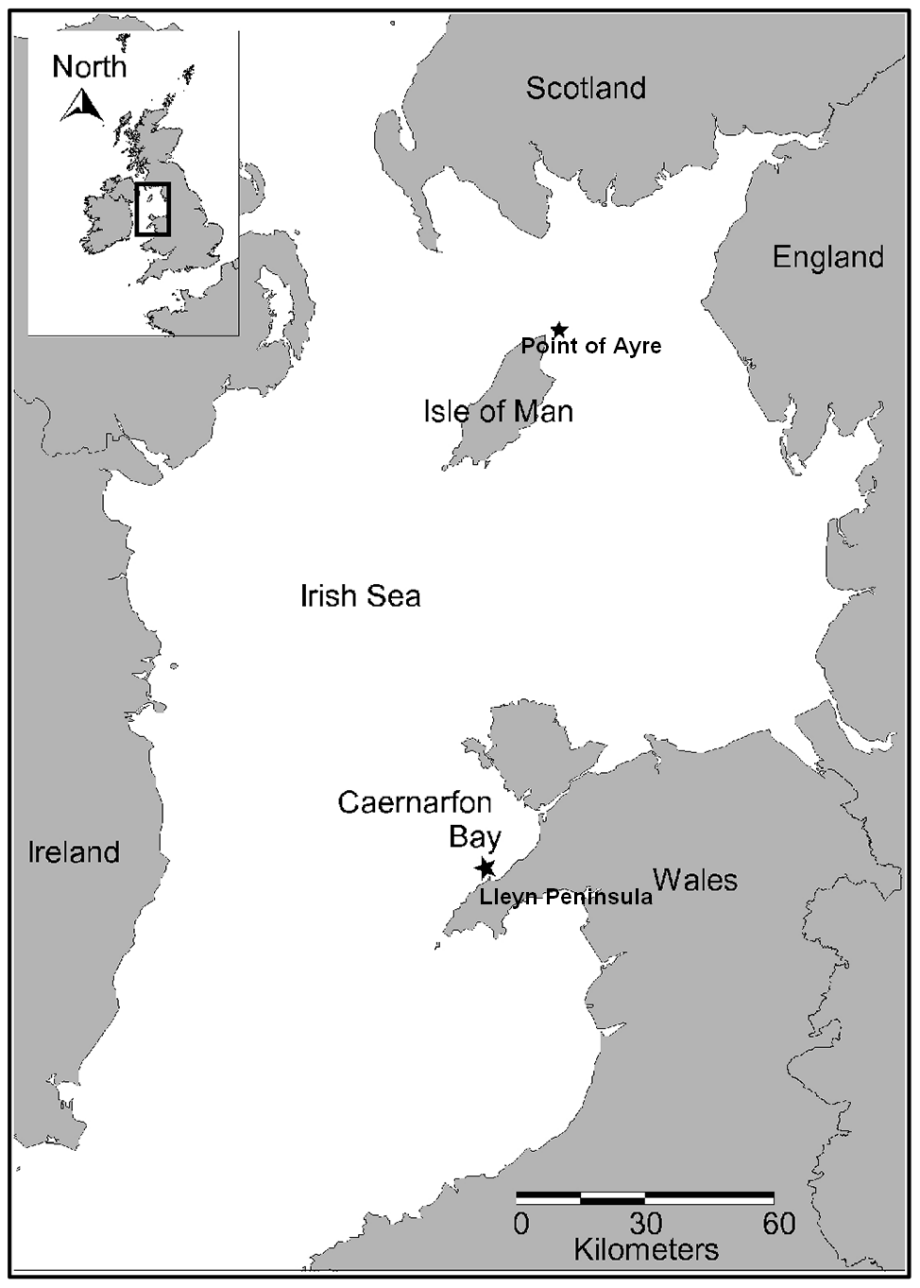
Study sites. Stars indicate *Modiolus modiolus* bed study sites north of the Point of Ayre (Isle of Man) and north of the Lleyn Peninsula in Caernarfon Bay (Wales).

**Figure 2:**
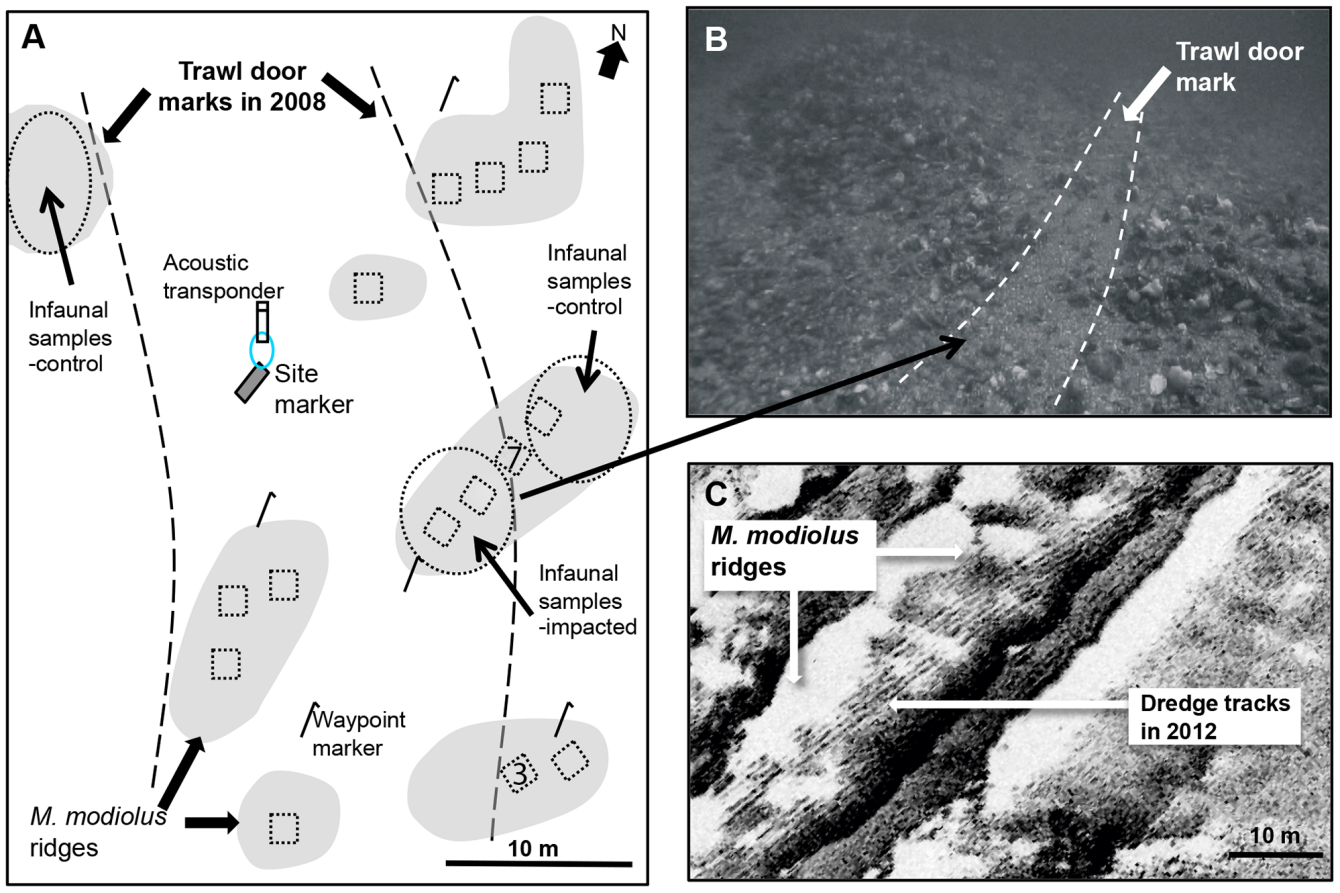
Details of study sites. (A) Map of fixed quadrat locations (dotted squares) on raised ridges (grey polygons) at Point of Ayre study site. Dotted ellipses indicate infaunal sample areas for impacted and control treatments. Two trawl door marks in 2008 are indicated by dashed lines. One trawl door mark in (A) is visible in the video-grab image (B) where the more extreme impact (compared to the net) in the path of the trawl door is also illustrated with dashed lines. The numbers “7” and “3” in (A) are quadrat numbers refered to in Figure 3 and Discussion (respectively). Metal waypoint pins enabled navigation around the site. (C) Side scan sonar image from 2012 at the study site off the north of the Lleyn Peninsula: marks from two gangs of scallop dredges are visible across the surface of the *Modiolus modiolus* ridges.

Previous survey data and side scan sonar outputs [34] were used to establish a study site in an extensive area of *Modiolus modiolus* bed 4.5 km off the Point of Ayre (Isle of Man; 54°26′.20N 004°18′.18W; Figure 1 & 2A). A steel marker with a hydroacoustic beacon (Sonardyne, Yateley, Hampshire, UK) was used for relocation and a corresponding hand-held hydroacoustic relocation device was used in conjunction with a compass to map the site to within 0.1 m accuracy (Figure 2A).

Side scan sonar imagery (Figure 2C) was used to identify an impact study site in June 2012 on another *M. modiolus* reef 5 km north of the Lleyn Peninsula (North Wales; 52 56′.99N 004°38′.56W; Figure 1 & 2C). Scallop dredging vessels had been recorded in the area during the preceding season (November 2011–April 2012) and the marks had not been recorded in all previous annual side scan sonar surveys.

The Point of Ayre (PoA) and north Lleyn Peninsula (nLP) sites both contained raised reef structures (1 m+) and high densities of *M. modiolus* (>350 m^−2^ see (9), [13] and present study). PoA and nLP were 33 and 30 m below chart datum with peak tidal flows of 1 ms^−1^ and 1.25 ms^−1^ respectively and both were fully saline [35], [36].

### Records and samples

Divers systematically filmed the 25 cells that made up 0.25 m^2^ quadrats at close range (<0.5 m) using high-definition handheld colour video-cameras (quadrats were then removed from the site).

At the PoA quadrat records were made between August and September in 2007, 2008 and 2009 at 12 positions relocated using fixed plastic pins on top of ridges of *M. modiolus*. In 2008 notification of the survey and a position was given to local shipping and fishing organisations in a Notice to Mariners a week before the survey. During the subsequent 2008 survey, 6 out of the 12 original quadrat positions were found to be impacted by a pair of clearly visible (to the diver) parallel furrows and a ‘swept’ area between that was tangential to the ridges of the natural bedform (Figure 2A & B) and consistent in size and orientation with the passage of an otter trawl. It is therefore likely that this occurred in response to the pre-survey information released.

Recording was conducted in a similar way at nLP in July 2012, except that quadrats were randomly placed in areas with conspicuous dredge marks and adjacent un-dredged areas.

**Figure 3:**
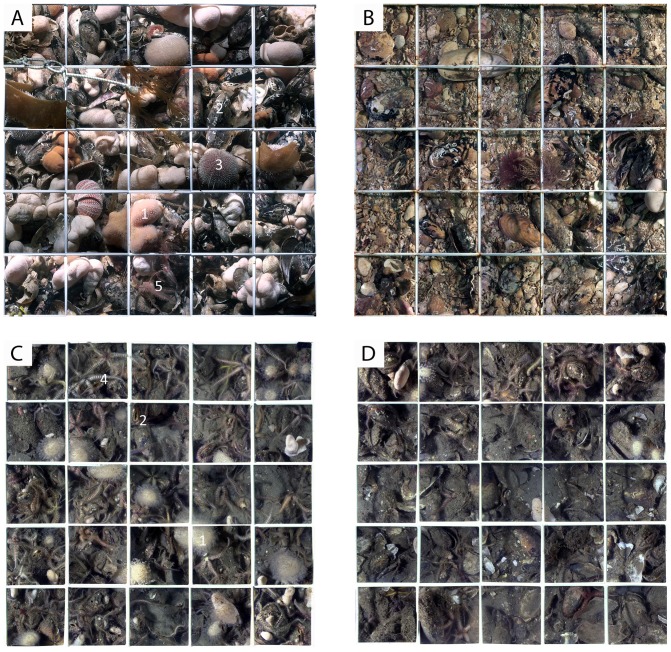
Mosaic quadrat images of quadrats. Quadrat 7 (indicated in Figure 2) from Point of Ayre in 2007 (A) and 2009 (B). (C) Unimpacted quadrat and (D) impacted quadrat from N. Lleyn Peninsula in 2012. Numbers indicate conspicuous epifauna: 1 *Alcyonium digitatum*, 2 *Modiolus modiolus*, 3 *Echinus esculentus*, 4 *Ophiothrix fragilis*, 5 *Antedon bifida*.

Frame grabs of each of the quadrat sub-cells were stitched together to create a high-resolution mosaic of the benthic community under each quadrat, from which conspicuous species were enumerated (Figure 3). Fauna were recorded to the highest taxonomic resolution possible and recording rules (file S1) were applied to reduce variability from cryptic organisms.

At PoA four random 0.0625 m^2^ infaunal samples were taken in 2009 from each of three *M. modiolus* ridge locations: Two outside of the marks recorded in 2008 where there was no evidence of trawl damage (Figure 2A: “Control”) and one where a ridge was found damaged in 2008 (Figure 2A: “Impacted”). Divers sampled to 20 cm depth and recovered material into 0.5 mm drawstring mesh bags. Samples were preserved in 5% buffered formaldehyde and sieved on a 0.5 mm mesh. Infaunal samples were sorted separately and all fauna identified to a coarse level of taxonomic resolution (Class level) sufficient to detect impacts [37]. Porifera, Hydrozoa, Anthozoa and Bryozoa were not used in subsequent analysis because they were better represented in the video analysis.

### Data treatment and statistical analysis

Multivariate analyses were conducted on Bray-Curtis similarity coefficients of square root transformed species abundance data, using PRIMER v6 with PERMANOVA+ software [38], [39]. Non-metric multidimensional scaling (MDS) was applied to Bray Curtis similarities using the Kruskal fit scheme [38] and, in the case of epifaunal data from PoA, a dummy variable was used to stabilise dispersion of sparse data [38]. Variation between impacted and unimpacted quadrats at the two sites were tested as fixed effects in one-way (nLP) and mixed two-way designs with year as random factor (PoA) using Permutational Multivariate Analysis of Variance (PERMANOVA) based on 9999 permutations and Type III sums of squares (SS). Type III SS is the most conservative SS method for PERMANOVA, fitting every term simultaneously and ensuring independence of all factors in unbalanced designs [39]. Within-site correlation differences through time in the PoA site were tested using PERMDISP (permutation of dispersion [39]). Taxa contributing to dissimilarities between treatments were investigated using a Similarity Permutation procedure (SIMPER; [38]).

Number of individuals (*N*), Shannon-Wiener's diversity (H′), Margalef's richness (d) and Pielou evenness (J) were imported into R (version 2.13.1, [41]) and tested for normality and heteroscedasticity. Effects of physical impact on diversity and evenness indices from quadrat records at both sites were tested (α of 0.05) by fitting linear mixed effects models (LMMs: lme4 package; [41]) with individual quadrats (both sites) and sampling year (PoA site) as random factors to account for spatial and temporal pseudoreplication. Impact (impacted vs non-impacted) was the categorical predictor (fixed factor) in the mixed model. Generalized LMMs with Poisson error distribution and logit link function were fitted to the abundance data (N; *M. modiolus* and epifauna) incorporating the same fixed and random factors as the LMMs [41] to cope with non-normal data in unbalanced, mixed-effect experiments [42]. Overdispersed Poisson models were refitted using Penalized Quasi Likelihood approximations (glmmPQL: MASS package [40]). The Akaike Information Criterion (AIC) was used to assess the effect of the physical impact on the null model for PoA and nLP while controlling for the random effects. Model selection was based on the lowest AIC score (Table S1). Infaunal count data from PoA cores conformed to the parametric assumptions and were therefore tested against impact treatments using standard one-way ANOVAs. All models were tested using residual plots to confirm that the assumptions of normality and sphericity of the residuals were met.

## Results

**Figure 4:**
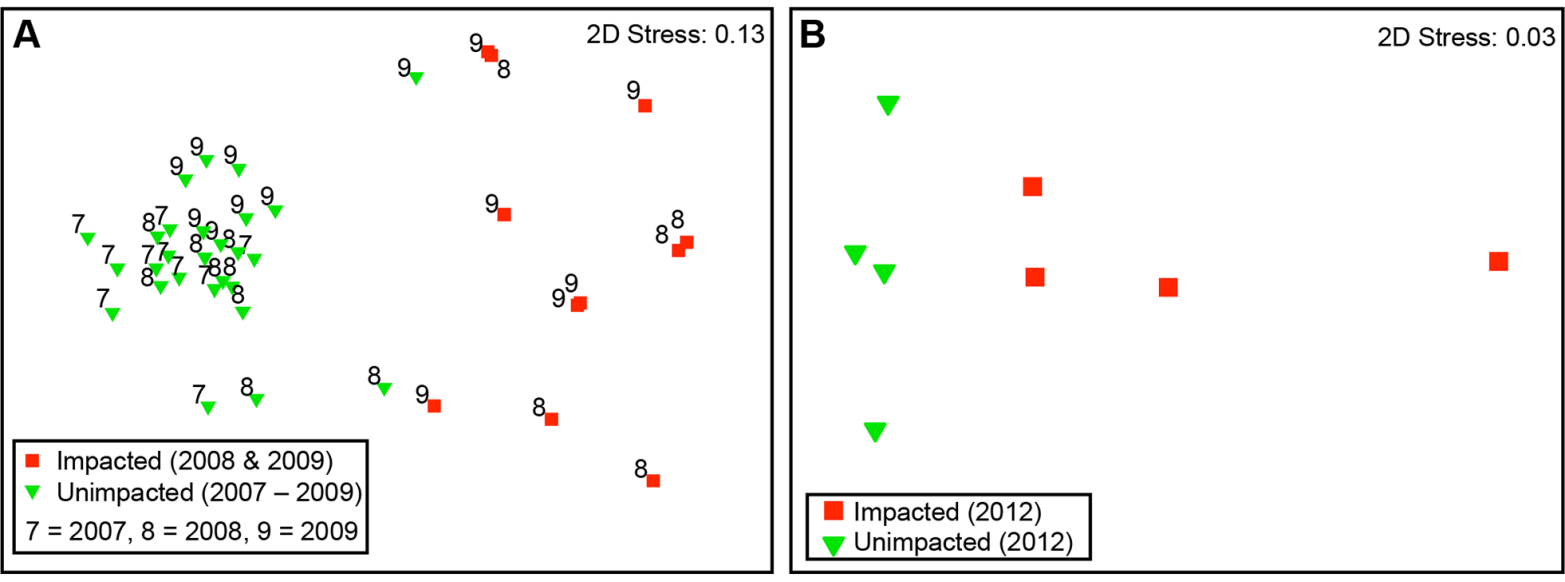
MDS plot showing the relationship between impacted and unimpacted epifaunal communities. (A) Point of Ayre. Dummy variable (present everywhere) used to create coherence in low abundance [impacted] data (see (36). (B) North of the Lleyn Peninsula.

In total 29 different taxa were recorded in video quadrats at the two study sites. At both sites there were significant impact effects on community composition (pseudo F = 24.37, p = 0.0001; pseudo F = 2.86, p = 0.03 for PoA and nLP respectively). There was also significant variability among years in the structure of the community at PoA (pseudo F = 2.52, p = 0.005). PERMDISP analysis indicated significant larger dispersion across time in epifaunal community samples following impact (deviations from centroid: F_(1,36)_ = 12.07; p<0.01). However, individual pairwise tests at PoA showed significant difference in dispersion occurred only after the trawling event in 2008 (2007 and 2008: t = 4.99; 2007 and 2009: t = 5.57; p<0.001) with no significant within site differences between 2008 and 2009 (t = 0.56; p = 0.69).The average dissimilarity between impact treatments at PoA site was high (85%) in the SIMPER analysis and driven by reductions in all but one (Paguridae) of the taxa in the impacted quadrat records. More than 90% of the average differences between unimpacted and impacted quadrats were accounted for by reductions in *Alcyonium digitatum* (L.), Actinaria, *Antedon bifida* (Pennant), Hydrozoa and *Modiolus modiolus* (SIMPER). At nLP the impact was less pronounced with 31.3% average dissimilarity between impacted and unimpacted treatments and reductions in the abundance of *Modiolus modiolus*, *Alcyonium digitatum*, *Ophiothrix fragilis* (Abildgaard), *Ascidiella* sp., *Flustra foliacea* (L.), *Pyura* sp. and Anomiidae accounting for 57% of the dissimilarity between treatments (SIMPER). Some encrusting and low-lying taxa at nLP were more abundant in records from impacted quadrats because upright emergent epifauna had reduced and revealed them (e.g. increased *Crisia eburnea* (L.) contributed 5.5% to dissimilarity). Overall, for both *M. modiolus* reefs there was compelling evidence of physical impact on the epifaunal communities (Figure 3 and 4) and the significant differences in dispersion between 2007 and 2009 at PoA indicated no recovery.

**Figure 5:**
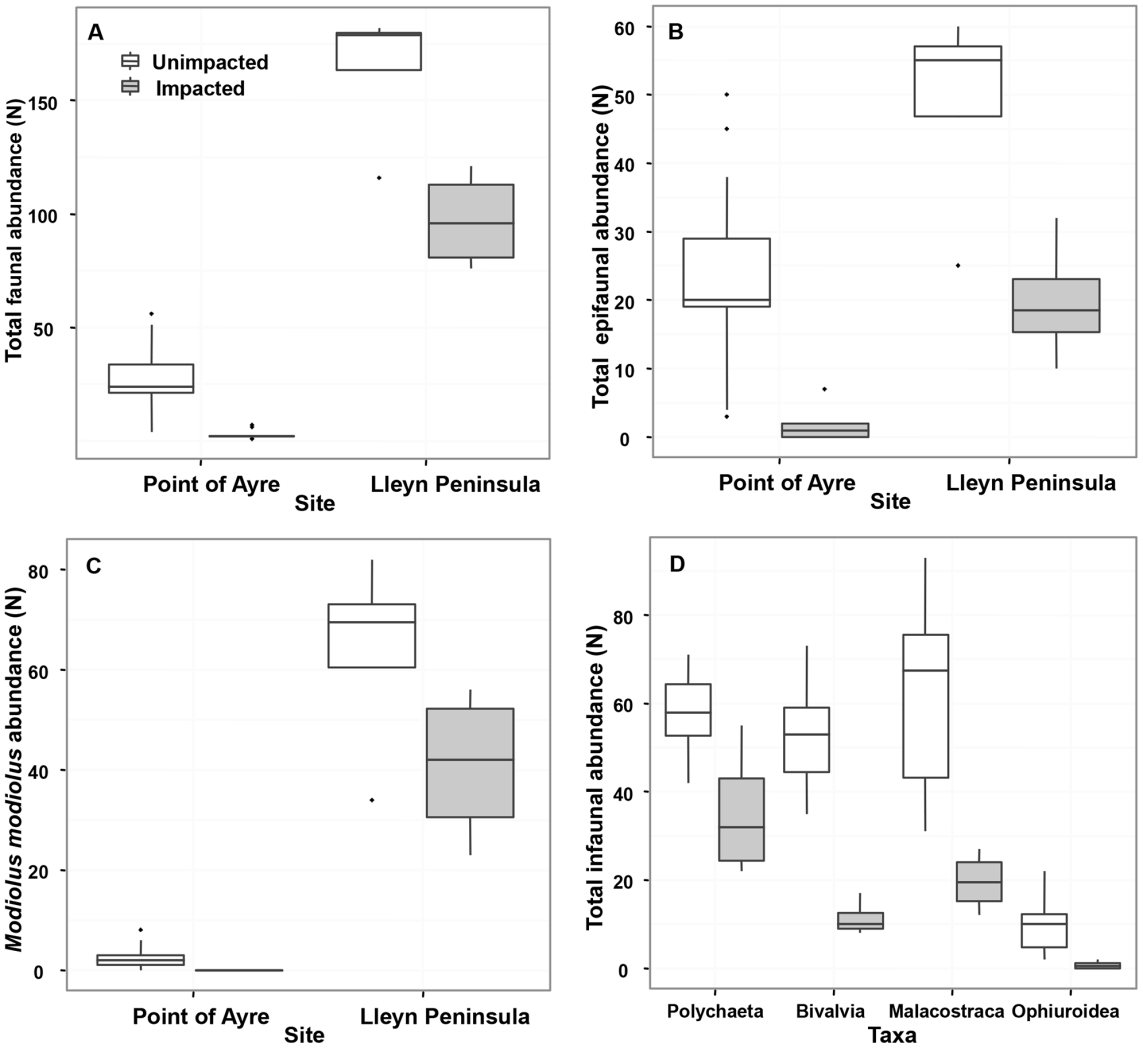
Reductions in epifauna and infauna following bottom-towed fishing gear. Total number of individuals (A), upright emergent epifauna (B) and numbers of *M. modiolus* (C) recorded on impacted and unimpacted 0.25×0.25 m video quadrats off Point of Ayre(PoA) and North Lleyn Peninsula (nLP). (D) Abundance of infaunal taxa contributing the most to the dissimilarities between impacted and unimpacted treatments at the PoA site (SIMPER). Box plots represent inter-quartile range, median, maximum and minimum values. The effect of physical impact was significant at α of 0.05 for all measures except *M. modiolus* abundance at nLP (Table 1).

In video quadrat data from PoA, significant reductions in number of individuals (N), numbers of upright emergent epifauna and total numbers of visible *M. modiolus* occurred at the impacted areas (Figure 5; Tables 1 and 2). Species richness (Margalef's d) and Shannon-Wiener's diversity (H′) and community evenness (J) were significantly lower in impacted quadrats (Figure 5; Tables 1 and 2). Overall, mean number of total individuals (N) was significantly reduced by 90.3% in trawled quadrats (2.63±1.96) compared to untrawled quadrats (27±12.23) (GLMM: t = −11.41; p<0.001). Most of the variation in N in impacted and unimpacted quadrats occurred between quadrat locations (σ^2^site = 0.39), varying little between years (σ^2^year = 0.09). At nLP there was a 59% lower mean abundance of total individuals (N) in video records from the scallop dredged areas (97.3±21.7 compared to 164±32.0; LMM) = 3.42; d.f. = 6; p<0.05). Lower abundances of *M. modiolus* and total upright emergent epifauna (mostly *A. digitatum* and *F. foliacea*) in dredged areas were significant only for the latter (LMM *M. modiolus* t = 1.75; p = 0.13; upright emergent epifauna t = 3.06; p<0.05; Figure 5). Shannon-Wiener's diversity (H′), Margalef's d richness and eveness (J) of the associated community were not significantly altered by impact (H′: t = −1.74, p = 0.13; d: t = −1.55, p = 0.17; J: t = −1.14, p = 0.29;).

**Table 1:** Abundance and diversity parameters measured for impacted and unimpacted *M. modiolus* communities.

	Total abundance (N)	Margalef's richness (d)	Shannon-Wiener's diversity (H′)	Pielou's evenness (J)	Epifaunal abundance	*M. modiolus* abundance
* **nLP**						
**Unimpacted**	164.0±32.0	1.3±0.3	1.3±0.1	0.6±0.0	48.8±16.0	63.8±20.7
**Impacted**	97.2±21.7	1.8±0.6	1.5±0.2	0.7±0.1	19.7±9.2	40.7±15.4
* **PoA**						
***2007***	33.6±13.6	1.8±0.2	1.5±0.2	0.8±0.1	29.0±12.0	2.1±1.3
***2008***						
**Unimpacted**	23.8±10.6	1.6±0.3	1.4±0.2	0.8±0.1	21±13	1.6±1.3
**Impacted**	1.8±0.4	0.7±0.8	0.3±0.4	1.0±0.0	0.6±0.9	0.0±0.0
***2009***						
**Unimpacted**	22±9.4	1.6±0.4	1.4±0.2	0.8±0.07	18±12	2.8±1.2
**Impacted**	3.3±2.5	1.0±0.7	0.5±0.5	0.9±0.2	2.2±0.9	0.0±0.0

* North Lleyn Peninsula (nLP) and Point of Ayre (PoA).

Mean +/− standard deviation values given.

**Table 2:** GLMM coefficients for diversity measures for impacted and unimpacted *M. modiolus* communities from PoA.

Response	Random effects	Intercept		Effect of disturbance (unimpacted vs impacted)
***Total abundance (N***				
	σ^2^year = 0.09	Estimate	3.24±0.1	−2.24±0.20
	σ^2^site = 0.39	t-value	32.51	−11.41
	σ^2^residual = 0.7	p-value	<0.001	<0.001
**M. modiolus ** ***abundance***				
	σ^2^year = 1.23×10^−10^	Estimate	0.38±0.16	−28.68±0.3
	σ^2^site = 0.83	t-value	2.33	−94.10
	σ^2^residual = 5.9×10^−10^	p-value	<0.05	<0.001
***Epifaunal abundance***				
	σ^2^year = 3.76×10^−6^	Estimate	3.10±0.11	−2.67±0.41
	σ^2^site = 0.85	t-value	27.22	−6.59
	σ^2^residual = 1.86	p-value	<0.001	<0.001
***Shannon-Wiener's diversity (H′)***				
	σ^2^year = 1.2	Estimate	1.48±0.06	−1.06±0.11
	σ^2^site = 0.3	t-value	25.66	−9.97
	σ^2^residual = 0.002	p-value	<0.001	<0.001
***Margalef's richness (d)***				
	σ^2^year = 8.66×10^−6^	Estimate	1.68±0.09	−0.8±0.18
	σ^2^site = 0.46	t-value	18.92	−4.49
	σ^2^residual = 0.002	p-value	<0.001	<0.001
**Pielou's evenness (J)**				
	σ^2^year = 1.42	Estimate	0.82±0.02	0.11±0.043
	σ^2^site = 0.09	t-value	44.47	2.46
	σ^2^residual = 0.0004	p-value	<0.001	<0.05

Fixed factor = physical impact; random factors = time (year) and quadrat position. Estimate includes ± standard deviation (sd). Significance at α = 0.05.

**See also Table S1**.

Using low taxonomic resolution 19 broad groups were recorded from infaunal samples at PoA. The trawled infaunal community in 2009 varied significantly from the two control sites (PERMANOVA: pseudo F = 9.02, p = 0.002) a year after the impact was first observed. In the SIMPER analysis, reductions in the abundances of bivalves, malacostracans, ophuroids and polychaetes accounted for 60% of the average differences between impacted and unimpacted samples. Each of these reductions in abundance was significant (Figure 5D; ANOVA:Polychaeta, F_(2,9)_ = 9.69, p<0.01; Bivalvia, F_(2,9)_ = 24.75, p<0.001; Malacostraca, F_(2,9)_ = 6.52, p<0.05; Ophiuroidea, F_(2,9)_ = 11.44; p<0.01).

## Discussion

The present study investigated the effects of single passes of bottom-towed fishing gear on rare protected *Modiolus modiolus* reef communities. The null model was rejected because there were substantial declines in the abundance of epifauna in response to both trawl and scallop dredges as well as declines in all major taxonomic groups in the infaunal community at the trawled site. The present study provides the most direct evidence yet of physical impacts on the community associated with this type of complex habitat. Abrasion of epifauna is undoubtedly one mechanism responsible for the changes observed but loss of structure formed by *M. modiolus* and the role that the species plays in pelagic-benthic coupling also probably account for reductions in most taxonomic groups (especially at PoA). The post impact increase in Paguridae at PoA is consistent with increased scavenging in other fishing gear impact studies [43], [44]. The results are also consistent with indirect studies elsewhere in the world where *M. modiolus* and associated epifaunal declines have been documented in dredging and trawling grounds [24], [25], [45], [46] and where *M. modiolus* as a species (not forming biogenic structures) has been shown to decline in experimentally trawled areas [28]. Similarly, other biogenic reefs formed by oysters *Ostrea chilensis* and horse mussels *Modiolus areolatus* in the Faveux Strait (New Zealand) shown widespread reductions in the associated community and reef habitat following prolonged dredging [26]. Overall, it would seem that all complex temperate biogenic habitats such as shellfish reefs, maerl, sea grass beds and bryozoans reefs shown declines in response to dredge and trawls [2], [26], [32], [47], [48].

### First-pass impacts

The horse mussel reef off the north Lleyn Peninsula has existed for at least 150 years [10] probably because it has traditionally been distant from Irish Sea demersal (bottom-towed) fishing ports and since the late 1990s protected in an MPA and by a fisheries by-law [49], [50]. At the Point of Ayre, a combination of strong currents, a busy shipping lane and unsuitable habitat has left the study site largely un-fished and recent vessel monitoring data confirmed that the area was “non-impacted seabed” [51]. The present studies are therefore on relatively un-impacted, if not pristine sites and document the substantial impact of single first passes of bottom-towed fishing gear. The video method of recording, which clearly favours epifauna, undoubtedly underestimated the severity of the impact where multi-layered epifauna have been largely removed over the majority of the substratum (PoA). There were instances where one or two species of understory turf were revealed and (erroneously) appeared to increase with impact (nLP). Didemnids, Bryozoa and Porifera all showed such increases at nLP, contributing 23% to dissimilarity between impacted and unimpacted quadrats in SIMPER analysis and probably contributed to a lack of significant change in diversity measures at nLP compared to PoA (Table 1). Without understanding the detailed structure of the understory this effect cannot be corrected or accounted for. Overall, the reductions of 90 and 59% of total epifaunal numbers supports the view that the majority of benthic impact occurs the first time an area is fished (e.g. [32], [52]) but also appears far greater in magnitude than modelled epifaunal biomass lost to fishing in surrounding Irish Sea habitats (8%: [53]). Given the sensitivity of the habitat, modern-day *M. modiolus* reefs are likely to be relics of their pre-fishing distribution. If so, then contemporary reference conditions for un-impacted benthic systems [53] may not account for these long-lived structure forming species in a classic ‘shifting baselines’ sense [54] and the bioengineering *M. modiolus* may well have occurred, albeit in lower densities, over more wide-spread areas as seen in other Atlantic studies [28], [46].

The magnitude of changes in the present study are similar to the differences in fauna between ridge and trough structures in naturally occurring beds (62%: [13]; [14]). In essence, the physical impact from bottom - towed gear removed ridge structure and appeared to reduce the community to a ‘trough’ habitat (*sensu* [14]) at PoA and, although declines in *M. modiolus* were not significant at nLP, clump structures were visibly flattened as well as showing significant epifaunal declines. The scale of change in epifaunal abundance, in particular, may be a useful indicator of condition in MPAs (EC Habitats and Species Directive; Marine (Scotland) Act 2010) but also Good Ecological Status (GES) under the EC Marine Strategy Framework Directive, where the biodiversity of ‘special’ habitats such as *Modiolus modiolus* reefs are currently being considered [15]. Large changes in the variance (see PERMDISP results) associated with epifaunal abundances might be expected across a reef experiencing low levels of physical impact that only cover part of it, whereas a substantial significant decline in mean epifaunal abundance (60% or more) would be expected across a reef experiencing fishing throughout; both scenarios would be incompatible with GES.

### Recovery and destabilisation

There was no evidence of recovery a year after impact was first recorded at Point of Ayre and since the long-lived structure forming species, *M. modiolus* (up to 48 years [55]), had significantly declined, this is unsurprising. Restorative experiments on *M. modiolus* beds have recently shown that semi-natural communities can recover within a year, but only when *M. modiolus* is translocated back into the habitat [56]. Irrespective of impact, there was also a change in the epifaunal community over time where *Alcyonium digitatum* and *Antedon bifida*, in particular, decreased in abundance. Although these observations probably reflect normal community dynamics they do highlight the need for investigations of the indirect effects of fishing gear such as re-suspended sediment [57] because large amounts of fine material are trapped in tidal areas under living *M. modiolus* reefs [4], [10]. It is possible that the mobilisation of this sediment could inhibit bivalve spat settlement [57] as well as the feeding of other species. Limited destabilisation of a ridge was observed beyond the initial impact in the present study where the tide appeared to have acted like wind in a sand-dune ‘blow-out’ (PoA, quadrat 3, 2009, Figure 2) and reduced the community to a crater next to a damaged section of reef.

### Scallop dredge vs trawl impact

On a heavily fished area of seabed in the Isle of Man it has been reported that otter trawls produced minimal bycatch and much less benthic damage compared to scallop dredges [58]: a contrast with the present study where the impact of the scallop dredge was not as great as the trawl. Although the present experimental design does not allow for direct comparison between fishing gears, it is nevertheless surprising that scallop dredges did not cause greater damage, given the metal dredge teeth at the leading edge of the gear. *M. modiolus* decline was not significant and evident physical structure remained in place post impact at nLP (Figure 3). It is plausible that dense *M. modiolus* reefs with mussel clumps [13], rapidly fill scallop dredges as bycatch and, affect the contact between the dredge teeth and the benthos (unlike a trawl door). Indeed, *M. modiolus* has been reported as a major component of an established scallop dredge fishery with 28% bycatch [59], but in a first-pass of fishing gear bycatch might be expected to be far greater when a dredge initially comes into contact with a reef. Overall, the levels of fishing disturbance to which the seabed has already been exposed may govern the impact of dredge gear.

### The dilemma of evidence-based conservation for rare habitats

To better understand the relative differences in gear impacts would require controlled, comparative fishing at similar sites. Such studies, however, are not ethically or legally possible when, as here, the protected biogenic habitat is rare and largely found within MPAs.

It is the authors' experience that the preclusion of premeditated impact studies on ethical and legal grounds can, paradoxically, be a significant obstruction to delivering the conservation objectives in MPAs because restrictions proposed by conservation managers can be challenged by stakeholders who perceive a lack of evidence of impact as evidence of absence of impact. Challenges to the adequacy of the evidence - base are apparent in the wider fisheries impact literature (eg [60] countered by [20] and [61] countered by [21]) and have also been used in political negotiation (e.g. [62]) but concerns about the evidence - base of conservation practice as a whole have also emerged (eg [17]). Calls for evidence-based conservation have acknowledged that conservation science lacks the resources to deliver meta-analyses in the same way as medical science [17], but limited scientific knowledge has long been used, for example, as an excuse to hinder the development of marine reserves [63]. In rare protected habitats where impact studies are unethical or illegal, the conservation manager is thus caught in an evidence trap when dealing with extant damaging commercial activities and no direct evidence of impact: a problem that is likely to be widespread and, ironically, unreported in the scientific literature.

## Supporting Information

File S1 **Video analysis recording rules.**
(DOCX)

Table S1 **GLMMs used to assess the effect of trawling impact on the PoA site.**
(DOCX)
