# Supplementary material for: The Substantial First Impact of Bottom Fishing on Rare Biodiversity Hotspots: A Dilemma for Evidence-Based Conservation
Source: PLoS One. 2013 Aug 14;8(8):e69904. doi: 10.1371/journal.pone.0069904 (PMC3743846; doi:10.1371/journal.pone.0069904)
Supplement: File S1 — Video analysis recording rules. (DOCX) [file pone.0069904.s001.docx]

### Supporting information S1. Video analysis recording rules

In order to improve the precision of records from videos of quadrats, rules were applied to the process. *Alcyonium digitatum* (L.), for example, were only counted when colonies were >15 mm because it was judged that colonies over this size could be consistently detected. *Modiolus modiolus* (L.) were counted if the mussels were clearly alive i.e. gaping and the mantel visible: this prevented largely subjective differentiation between live and dead shells. For sponges, hydroids and bryozoans, erect colonies > 10 mm long such as *Nemertesia antennina* (L.) and *Flustra foliacea* (L.) were counted, again because colonies smaller than this could not be reliably recorded.
