# Supplementary material for: The Substantial First Impact of Bottom Fishing on Rare Biodiversity Hotspots: A Dilemma for Evidence-Based Conservation
Source: PLoS One. 2013 Aug 14;8(8):e69904. doi: 10.1371/journal.pone.0069904 (PMC3743846; doi:10.1371/journal.pone.0069904)
Supplement: Table S1 — GLMMs used to assess the effect of trawling impact on the PoA site. (DOCX) [file pone.0069904.s002.docx]

Table S1. GLMMs used to assess the effect of trawling impact on the PoA site.

| **Response variable** | **Fixed factors** | **Random factors** | **AIC** | **Deviance** | **Loglik** | **R^2^** |
| --- | --- | --- | --- | --- | --- | --- |
| Total abundance (N) | impact | quadrat+year | 140.4 | 132.4 | -66.2 | 0.94 |
|  | Null | quadrat+year | 228.0 | 222.0 | -111.0 | 0.002 |
| *M. modiolus* abundance | impact | quadrat+year | 50.9 | 42.9 | -21.5 | 0.99 |
|  | Null | quadrat+year | 76.3 | 703.0 | -35.1 | 0.001 |
| Erect epifauna abundance | impact | quadrat+year | 141.6 | 133.6 | -66.8 | 0.95 |
|  | Null | quadrat+year | 242.9 | 236.9 | -118.5 | 0.002 |
| Shannon-Wiener's diversity (H') | impact | quadrat+year | 29.4 | 12.8 | -9.7 | 0.78 |
|  | Null | quadrat+year | 67.3 | 58.2 | -29.7 | 0.004 |
| Margalef's Richness (d) | impact | quadrat+year | 58.6 | 43.9 | -24.3 | 0.48 |
|  | Null | quadrat+year | 71.4 | 61.3 | -31.7 | 0.004 |
| Pielou's evenness (J) | impact | quadrat+year | -42.5 | -63.3 | 26.3 | 0.20 |
|  | Null | quadrat+year | -43.8 | -57.6 | 25.9 | 0.13 |

Akaike’s Information Criterion (AIC), model deviance and Log-likelihood (Loglik) are used as proxies of model fitness. R^2^ is the variance explained by the GLMMs.
